# Extended negative pressure wound therapy-assisted dermatotraction for the closure of large open fasciotomy wounds in necrotizing fasciitis patients

**DOI:** 10.1186/1749-7922-9-29

**Published:** 2014-04-15

**Authors:** Jun Yong Lee, Hyunwook Jung, Ho Kwon, Sung-No Jung

**Affiliations:** 1 Department of Plastic and Reconstructive Surgery, Incheon St. Mary’s Hospital, The Catholic University of Korea, 56, Dongsu-ro, 403-720 Bupyeong-gu, Incheon, South Korea; 2 Department of Plastic and Reconstructive Surgery, Uijeongbu St. Mary’s Hospital, The Catholic University of Korea, 271 Cheonbo-ro, 480-717, Uijeongbu-si, Gyeonggi-do, South Korea

**Keywords:** Necrotizing fasciitis, Negative pressure wound therapy, Dermatotraction, Fournier’s gangrene, Fasciotomy

## Abstract

**Background:**

Necrotizing fasciitis (NF) is a rapid progressive infection of the subcutaneous tissue or fascia and may result in large open wounds. The surgical options to cover these wounds are often limited by the patient condition and result in suboptimal functional and cosmetic wound coverage. Dermatotraction can restore the function and appearance of the fasciotomy wound and is less invasive in patients with comorbidities. However, dermatotraction for scarred, stiff NF fasciotomy wounds is often ineffective, resulting in skin necrosis. The authors use extended negative pressure wound therapy (NPWT) as an assist in dermatotraction to close open NF fasciotomy wounds. The authors present the clinical results, followed by a discussion of the clinical basis of extended NPWT-assisted dermatotraction.

**Methods:**

A retrospective case series of eight patients with NF who underwent open fasciotomy was approved for the study. After serial wound preparation, dermatotraction was applied in a shoelace manner using elastic vessel loops. Next, the extended NPWT was applied over the wound. The sponge was three times wider than the wound width, and the transparent covering drape almost encircled the anatomical wound area. The negative pressure of the NPWT was set at a continuous 100 mmHg by suction barometer. The clinical outcome was assessed based on wound area reduction after treatment and by the achievement of direct wound closure.

**Results:**

After the first set of extended NPWT-assisted dermatotraction procedures, the mean wound area was significantly decreased (658.12 cm^2^ to 29.37 cm^2^; *p* = 0.002), as five out of eight patients achieved direct wound closure. One patient with a chest wall defect underwent latissimus dorsi musculocutaneous flap coverage, with primary closure of the donor site. Two Fournier’s gangrene patients underwent multiple sets of treatment and finally achieved secondary wound closure with skin grafts. The patients were followed up for 18.3 months on average and showed satisfactory results without wound recurrence.

**Conclusions:**

Extended NPWT-assisted dermatotraction advances scarred, stiff fasciotomy wound margins synergistically in NF and allows direct closure of the wound without complications. This method can be another good treatment option for the NF patient with large open wounds whose general condition is unsuitable for extensive reconstructive surgery.

## Introduction

Necrotizing fasciitis is a rapid progressive infection of the subcutaneous tissue or fascia that usually occurs in the groin and lower extremities [1]. Early diagnosis with prompt surgical debridement is essential in management of this rapidly progressing disease [2,3]. However, even after proper management to control infection, a large open wound usually remains; to cover this, surgical intervention such as skin graft, local flap, or free flap is required [4-6]. A delay in coverage of this residual open wound may result in delayed infection, debilitating patient condition, and even generalized sepsis. However, surgical options are often limited as poor patient condition restricts the use of time-requiring extensive surgeries such as local flap or free flap coverage. Skin grafting usually requires a long time to heal, as the wound bed is often dirty and unstable. Negative pressure wound therapy (NPWT) has been used to control chronic wounds as it increases tissue perfusion and decreases wound edema [7]. Although NPWT can improve a wound’s condition, it cannot close it completely, so other operations are required for wound coverage [8]. Dermatotraction is a surgical option that gradually approximates the margins of large wounds with a traction device. Successful dermatotraction can close fasciotomy wounds directly, and may restore the function and appearance of the fasciotomy wound site. Dermatotraction has been used to close open fasciotomy wounds in compartment syndrome [9,10]. Whereas the fasciotomy wound in compartment syndrome is supple, the fasciotomy wound in necrotizing fasciitis is usually scarred, and stiffer than the wound in compartment syndrome due to prolonged wound preparation. Dermatotraction in the necrotizing fasciitis patient may therefore be ineffective, and the traction can disturb circulation in the stiff skin flaps, resulting in skin necrosis. Although it provides an attractive alternative for the necrotizing fasciitis patient in poor general condition, dermatotraction has remained an alternative surgical option to date. To decrease the likelihood of skin flap necrosis, and to facilitate skin flap mobilization for direct wound closure in the necrotizing fasciitis patient who had undergone dermatotraction, the authors applied extended NPWT over the dermatotraction device during treatment of the open wound. The authors present a report of clinical results of this practice, followed by a discussion of the clinical basis of extended NPWT-assisted dermatotraction in closing the large open wound of the necrotizing fasciitis patient.

## Materials and methods

The institutional review board of the Catholic University of Korea Uijeongbu St. Mary’s Hospital approved this study, a retrospective case series of adult necrotizing fasciitis patients who referred to the plastic surgery department for wound closure following open fasciotomies. Between 1 January 2005 and 31 December 2012, 15 patients were identified from medical records and patient lists. The inclusion criteria for necrotizing fasciitis patients were (1) abscess or gas bubbles along the fascial plane in computed tomography (CT) scan or magnetic resonance image (MRI) scans; (2) open fasciotomy performed immediately after initial diagnosis; and (3) manageable local and systemic infection. The exclusion criteria were (1) patients with cardiopulmonary failure, and (2) patients who could not cooperate the treatment plan due to uncontrolled mental disorder. All patients underwent periods of wound preparation by necrectomies and fasciectomies for infection clearance, and were then treated with extended NPWT-assisted dermatotraction for the closure of the resultant open wounds caused by necrotizing fasciitis. Eight patients (seven males and one female) were enrolled in this study. The mean age of the patients was 53.5 years (40–72). Three patients underwent open fasciotomies on their perineal areas; three underwent open fasciotomies on their lower extremities; two underwent open fasciotomies on their trunks. Seven out of eight patients had underlying co-morbidities and five patients had diabetes mellitus.

Before we performed dermatotraction, we prepared the fasciotomy wound with thorough debridement and irrigation. After the wound preparation, we applied elastic vessel loops (SURGI-LOOP®, Scanlan, Minnesota, USA) on both wound margins in a shoelace manner. We anchored the vessel loops using skin staples one to two centimeters away from the skin margin so as not to compromise the skin flap’s marginal circulation. When approximating the skin margins, we pulled the vessel loops until the capillary refills of the skin margins disappeared. After sustaining traction for 10 minutes, we evaluated the capillary refills of the skin flaps. If there was sustained absence of capillary refill, we released the vessel loops to relax both skin margins by about one to two centimeters. Then we repeated the capillary refill examination until the skin flaps were approximated maximally by vessel loop traction while retaining the proper capillary refills of the both skin flap margins. Then we covered the dermatotraction-applied fasciotomy wounds with an extended NPWT device. We applied a sponge three times larger than the width of the wound to decrease edema, to increase tissue perfusion, and to facilitate both skin flaps’ mobilization. We applied transparent surgical drapes over the NPWT sponge so that it almost encircled the anatomical area of the fasciotomy. We set the negative pressure of the NPWT device at a continuous 100 mmHg by suction barometer. We changed the NPWT device every second or third day and simultaneously readjusted the tension of dermatotraction.

For the patients who achieved tension-free skin margin approximation after the treatment, the fasciotomy wounds were closed directly with sutures. If there were residual skin defects that could not be closed directly due to marginal tissue loss by debridement, the defects were covered with split-thickness skin grafts (STSG) or local flaps.

The clinical outcome was assessed by wound area reduction after the treatment, and by achievement of direct closure of the fasciotomy wound. The paired *t*-test was used to compare the wound areas before and after the treatment using SPSS 12.0 (IBM, New York, USA). We considered *p* values less than 0.05 statistically significant.

## Results

Patient demographics and clinical results are summarized in Table 1. The mean wound preparation time was 32.4 days (6–46 days) to start NPWT assisted dermatotraction. The mean initial open wound area was 658.12 cm^2^ (160-1075 cm^2^), and this was significantly decreased to 29.37 cm^2^ (0-150 cm^2^, *p* = 0.002) after the first set of treatment, as five out of eight patients achieved direct wound closure. The mean extended NPWT-assisted dermatotraction treatment period was 16 days (5–40 days). There was no skin flap necrosis at the dermatotraction site. The patient with chest wall tissue defect was treated with latissimus dorsi musculocutaneous flap coverage, with minimized donor tissue harvest allowing primary closure of donor site. The Fournier’s gangrene patients who could not achieve direct wound closure underwent multiple sets of extended NPWT-assisted dermatotraction, and finally achieved wound closure by secondary closure with split-thickness skin grafts. The patients were followed up for 18.3 months on average (2–59 months). During the follow-up period, the patients who achieved direct wound closure showed satisfactory results without wound recurrence. Two patients showed focal infection signs; these were managed with antibiotic treatments. Although there was scar widening at the wound closure area, they were managed conservatively.

**Table 1:** Patient demographics and clinical results

**Patient no.**	**Sex**	**Age**	**Diagnosis**	**Wound preparation period**	**Wound area after wound preparation (cm**^**2**^**)**	**Wound area after the first set of extended NPWT assisted dermatotraction (cm**^**2**^**)**	**Extended NPWT assisted dermatotraction cycle**	**Extended NPWT assisted dermatotraction period**	**Final results**	**Complications requiring surgical interventions**	**Follow-up duration (months)**	**Co-morbidities**
1	Male	62	Necrotizing fasciitis, thigh and lower leg, Lt.	6	500 (50 × 10, thigh) 455 (35 × 13, lower leg) 80 (10 × 8, posterior calf)	0 (thigh, lower leg) 25 × 35 (posterior calf)	2	5	Direct closure, STSG (posterior calf)	None	59	None
2	Male	59	Necrotizing fasciitis, thigh, Rt.	46	825 (55 × 15)	0	4	14	Direct closure	None	4	DM, Pn, TB, Liver abscess
3	Female	72	Necrotizing fasciitis, buttock and thigh, Lt.	22 (thigh), 47 (buttock)	400 (40 × 10, thigh) 675 (45 × 15, buttock)	0	4 (thigh) 3 (buttock)	12 (thigh) 10 (buttock)	Direct closure	None	23	DM, CVA
4	Male	40	Necrotizing fasciitis, chest wall, Lt.	40	1000 (50 × 20)	0	14	40	Direct closure	None	27	HBV
5	Male	43	Necrotizing fasciitis, chest wall, Lt.	28	160 (20 × 8)	35 (7 × 5)	4	14	Latissimus dorsi musculocutaneous flap coverage	None	2	DM
6	Male	40	Fournier’s gangrene	22	450 (30 × 15)	0	7	23	Direct closure	None	2	TB
7	Male	53	Fournier’s gangrene	44	300 (20 × 15)	50 (10 × 5)	6	15	Direct closure	Partial wound dehiscence - secondary closure	24	DM, HTN, Paraplegia
8	Male	59	Fournier’s gangrene	39	500 (25 × 20)	150 (15 × 10)	3	6	Direct closure, STSG	Partial wound dehiscence - secondary closure with STSG	6	DM, HTN, CRF
Mean		54		32.4	658.12	29.37	5.4	16			18.3	

*Abbreviations*: DM diabetes mellitus, HTN hypertension, Pn pneumonia, TB tuberculosis, CVA cerebrovascular accident, CRF chronic renal failure, HBV hepatitis B, STSG split-thickness skin grafts.

### Case 1

**Figure 1:**
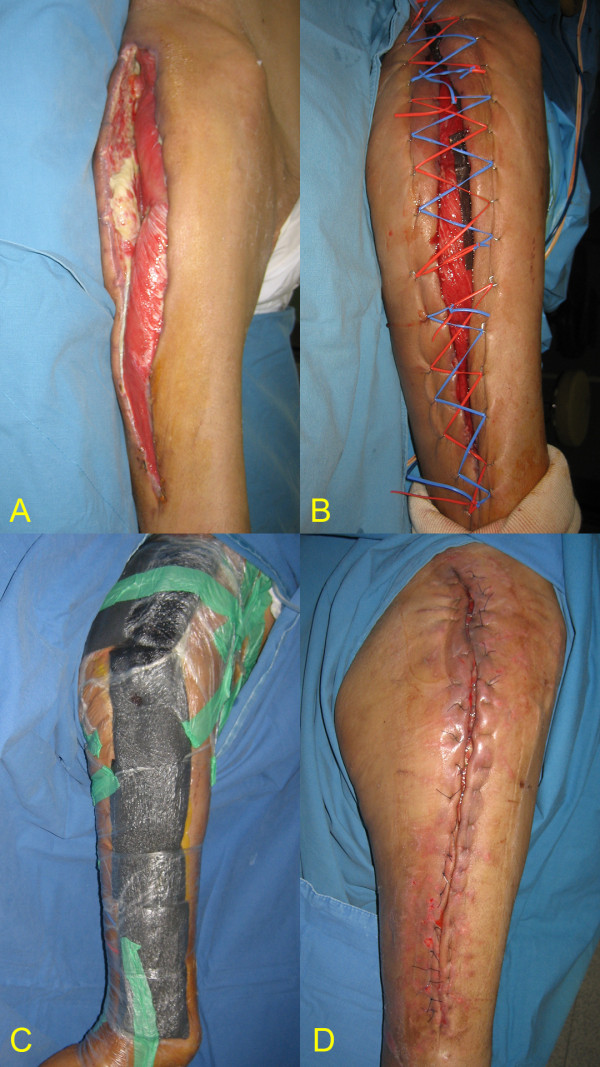
**Open fasciotomy wound closure with extended NPWT-assisted dermatotraction in necrotizing fasciitis; A 59-year-old male patient with necrotizing fasciitis on his right thigh showed contracted skin margins with necrotic tissues on the 14th day after initial fasciotomy. (A)**. After 46 days of wound preparation, the elastic vessel loop is applied for the dermatotraction in a shoelace manner **(B)**. The extended NPWT assisted the underlying dermatotraction in closing the open fasciotomy wound **(C)**. After the 14 days of treatment, the fasciotomy wound could be closed directly **(D)**.

A 59-year-old male patient had necrotizing fasciitis on his right thigh without a suspected initiating factor. The patient had been diagnosed with diabetes mellitus 20 years before. The general surgeons performed a fasciotomy on his left thigh with thorough debridement and wound irrigation. Two weeks after initial management, the patient was transferred to the plastic surgeon for wound coverage. The fasciotomy wounds spanned the lateral aspect of thigh to buttock with an area of about 55 × 15 cm; this was covered with granulation tissue. The exposed wound showed contracted skin margins with partially necrotic subcutaneous tissues and fascia (Figure 1A). After 46 days of wound preparation following initial fasciotomy, the patient underwent NPWT-assisted dermatotraction (Figure 1B, C). After 14 days of treatment, the fasciotomy wound could be closed directly (Figure 1D).

### Case 2

**Figure 2:**
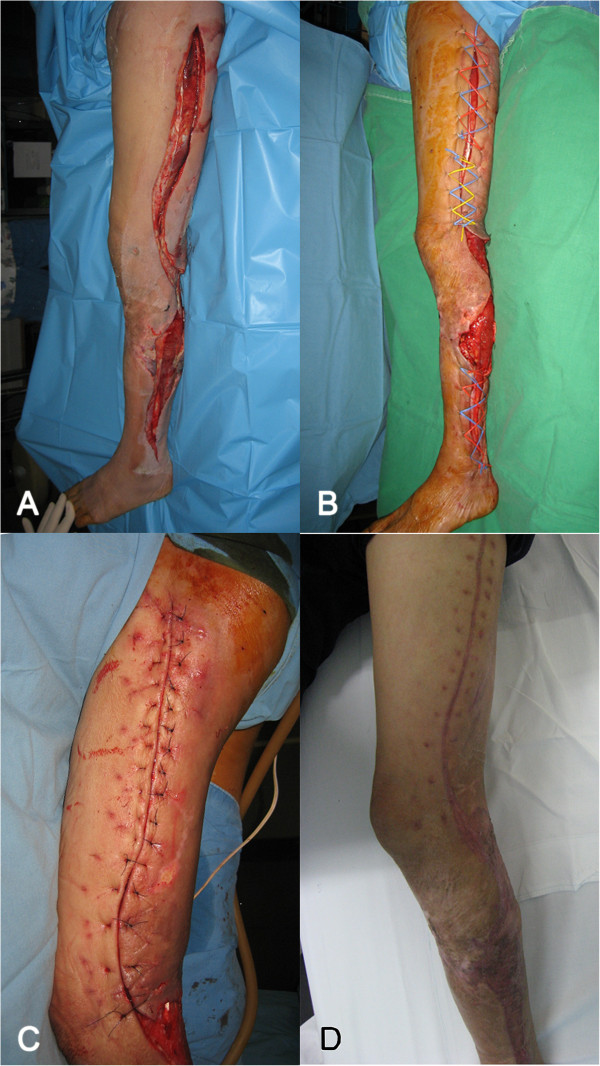
**Open fasciotomy wound closure with extended NPWT-assisted dermatotraction in necrotizing fasciitis; A 62-year-old male patient with necrotizing fasciitis on the left lower extremity underwent open fasciotomy on his thigh and lower leg. (A)**. After 7 days of thorough wound debridement and preparation, extended NPWT-assisted dermatotraction was applied **(B)**. After two cycles of treatment, the fasciotomy wounds were closed directly, and the posterior calf’s raw surface was covered with split-thickness skin graft **(C)**. Three months after wound closure, the wounds were completely healed without complications **(D)**.

A 62-year-old male patient developed painful swelling on his left thigh and lower leg without suspected initiating factors. The patient was transferred to our hospital antibiotic treatment at the local hospital failed. On admission, the patient showed bullae and swelling on the entire left lower extremity with concomitant ongoing necrosis on posterior calf skin. An MRI scan revealed necrotizing fasciitis of the entire left lower extremity. The patient underwent emergent open fasciotomy of lower extremity with debridement (Figure 2A). After seven days of thorough wound debridement and irrigation, the patient underwent two cycles of extended NPWT-assisted dermatotraction for the open fasciotomy wound closure (Figure 2B). Except for the necrosed posterior calf skin, which was covered with split-thickness skin grafts, the open fasciotomy wounds were closed directly without tension (Figure 2C). The patient was followed up at the outpatient department for 59 months, and there were no complications except for widening of the scar (Figure 2D).

### Case 3

**Figure 3:**
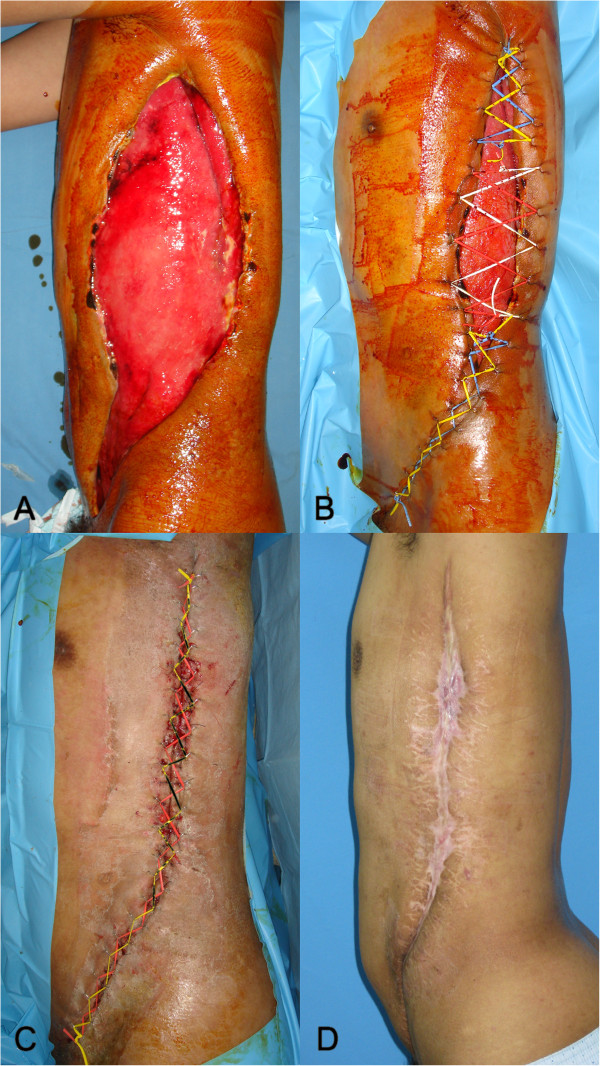
**Open fasciotomy wound closure with extended NPWT-assisted dermatotraction in necrotizing fasciitis; A 43-year-old male patient with necrotizing fasciitis that had developed an abscess in the left axilla underwent open fasciotomy one month before presentation. (A)**. After 40 days of wound preparation since initial fasciotomy, the patient underwent NPWT-assisted dermatotraction, which decreased the size of wound prominently after 6 days of treatment **(B)**. The wound was closed directly after 40 days of NPWT assisted dermatotraction **(C)**. The patient was followed up for 27 months and the wound was completely healed without complications **(D)**.

A 43-year-old male patient who was hepatitis B virus carrier developed necrotizing fasciitis that begun with an abscess in the left axilla. He was treated with serial surgical debridement at a local clinic for one month, but still had an open wound of 50 × 20 cm on his left trunk when he transferred to our department (Figure 3A). After thorough debridement and wound preparation for 40 days, we applied extended NPWT-assisted dermatotraction on his open wound. The wound had decreased prominently six days after initial application of the NPWT-assissted dermatotraction (Figure 3B). We were able to close the wound primarily without tension on 40 days of the treatment without infection (Figure 3C). The patient was discharged without complications five days after the closure. The patient was followed up regularly at the outpatient department, and there was no complication but a widened scar at 27 months (Figure 3D).

## Discussion

Necrotizing fasciitis is a rare, life-threatening condition that affects the limbs, groin, and trunk. It is a rapid, progressive infection of subcutaneous tissue and fascia that leads to thrombosis of cutaneous microcirculation and infection of soft tissues that can spread to the whole extremity in hours [11]. When diagnosis and treatment are delayed, the mortality rate can rise up to 70-100% [12-14]. As the infection progresses, tissue erythema darkens as the necrosis develops with bullae formation. Occasionally, tissue crepitus may be palpable during the course of the disease. Because the diagnosis is based primarily on clinical findings, early diagnosis of the necrotizing fasciitis is challenging [15]. Necrotizing fasciitis should be promptly recognized and aggressively surgically debrided along with prompt administration of broad spectrum antibiotics until the causative organism can be identified by cultures. The disease can be confirmed by surgical findings such as grayish necrotic deep fascia, a lack of resistance to blunt dissection, lack of bleeding of the fascia, and the presence of foul odor with pus [16]. Because necrotic fascia involvements are usually more widespread than the skin lesion, the surgical debridement must be extended to the viable tissue layers [17,18]. After early surgical debridement and systemic antibiotics treatment, serial wound follow-up should be continued. However, most necrotizing fasciitis patients have underlying diseases such as diabetes, peripheral vascular disease, or systemic immunosuppression [19]. These comorbid patients are apt to progress into severe infection or sepsis without coverage of the open wound.

Open fasciotomy wounds have several distinct characteristics to consider in planning an operative strategy. When body parts are simplified for fasciotomy, they can be substituted by assembles of cylinders standing for closed compartments. Fasciotomy is usually performed along one side of the longitudinal axis, perpendicular to the relaxed skin tension line. As fasciotomy releases all the retention forces and tissue pressures of the cylindrical compartment, the closed compartment can be effectively released, but this results in an open raw surface and diminished tissue pressure exposing underlying muscle or soft tissues. Moreover, the prolonged wound preparation period induces wound marginal contraction and wound margin inversion, which aggravate the wound widening and surrounding tissue edema. These wide-open raw surfaces are essential for a thorough wound debridement and infection clearance in the necrotizing fasciitis patient. For the wound closure of these large open wounds, skin grafting or local or free flap coverage should be used, although these result in suboptimal functional and cosmetic wound coverage.

**Figure 4:**
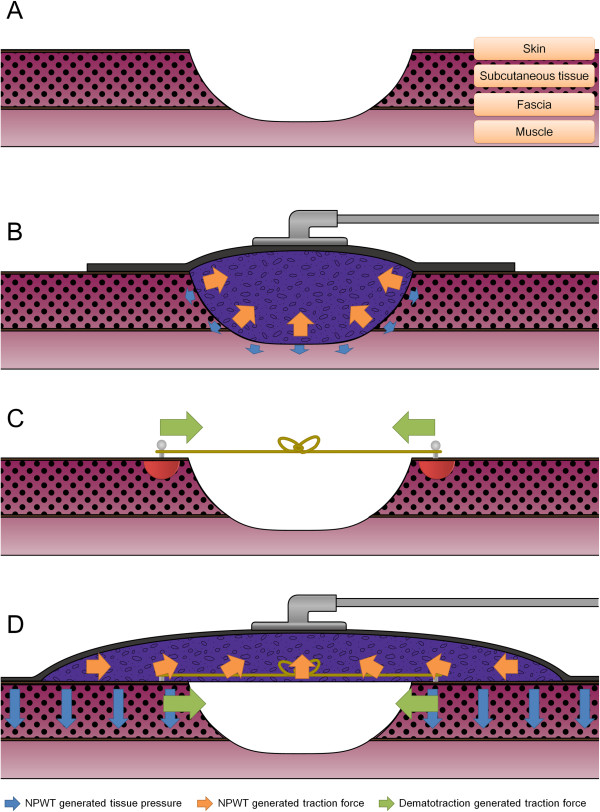
**Theoretical basis of extended NPWT-assisted dermatotraction; The fasciotomy releases all the retention forces by fascia and skin, thus decrease the tissue pressures of the cylindrical compartment. (A)**. The cavitary NPWT increases the tissue pressure with shallow penetration to the deep tissue, and limits wound contraction because of the intervening sponge **(B)**. The dermatotraction forces are concentrated on the anchoring point, which can disturb tissue perfusion and necrose the skin, especially in the stiff open fasciotomy wound of necrotizing fasciitis (red semicircle, **C**). Extended NPWT increases normal skin perfusion and sheers the wound margins to the central axis of the fasciotomy. This assists the dermatotraction by distributing the concentrated traction forces at the anchoring point and further approximating the wound margins. The near-circumferential adhesive surgical drape of the NPWT also limits tissue edema and delivers NPWT-generated increments of tissue pressure to the deep tissues like an elastic stocking **(D)**.

The authors developed treatment strategies in closure of the large open fasciotomy wound by reversing the fasciotomy wound-widening cascade. We think that restoration of the tissue pressure provided by fascia and skin is the key to closure of the open fasciotomy wound. Our primary treatment goal was to achieve effective tissue pressure, because, as with the pressure stocking, this decreases tissue edema and increases venous blood flow. Our secondary treatment goal was to approximate the wound margin for tension-free wound closure. Because these are large discharging open wounds, we utilized NPWT as a pressure device. Kairinos shows that tissue pressure increases with the amount of suction in NPWT [20]. However, this increased pressure penetrates no more than 1 mm into the tissue [21]. For deeper penetration, the surface area of applied pressure should be increased [22]. In NPWT dressing methods, a circumferential dressing was superior to a cavity dressing in increasing tissue pressure [20]. Despite this, we did not apply the sponge circumferentially because of the proximal location of the fasciotomy wound and the possibilities of distal circulatory compromise or venous congestion, as with the tourniquet. Instead, we extended the sponge three times wider than the open wound and extended the transparent adhesive surgical drape to nearly encircle the anatomical area of the fasciotomy for the NPWT. In this way, the surgical drape prevented edema by retaining the skin and conveying the traction forces by NPWT to the underlying tissues to increase tissue pressure. We also set an appropriate suction pressure to maximize tissue pressure while leaving blood perfusion of the underlying tissue undistrurbed. Although increasing suction pressure also increases tissue pressure [20] and maximizes wound fluid removal [23], it can decrease the perfusion of the underlying tissue [24], and may cause patient discomfort. At the wound edge, the microvascular blood flow can be maximized at as low a level as −80 mmHg of NPWT [25]. Maximum wound contraction can be achieved at −75 mmHg [23], so we continuously set the NPWT suction pressure at -100 mmHg (lower than the conventional −125 mmHg) to increase tissue pressure and wound fluid removal while maximizing wound contraction and microvascular blood flow. These extended NPWT methods act like a compression garment, applying a centripetal compression effect to increase tissue pressure. However, increased tissue pressure by extended NPWT reduced over 48 hours of application, as it was non-circumferential [20]. Moreover, the sponges in the wound cavity limited the wound contraction by the NPWT [26]. To approximate the longitudinal fasciotomy wound further, we applied the dermatotraction at both skin margins under the NPWT sponge. The dermatotraction vessel loop pull the both skin margins continuously, allowing stress relaxation of the contracted skin and preventing the NPWT sponge from filling the wound cavity, thus maximizing wound contraction by NPWT [26]. In this way, the dermatotraction acted as an elastic corset lacing. Skin necrosis by dermatotraction is usually caused by the concentration of traction forces at an anchoring point, which compromises skin perfusion. However, in extended NPWT-assisted dermatotraction, the NPWT on the normal skin increases the skin flap perfusion [27] and sheers the skin flap to the center of the contraction axis; this distributes the concentrated traction forces at the dermatotraction anchoring point to the skin flap (as shown in Figure 4). In this way, the dermatotraction effectively approximates both skin flaps, avoiding skin perfusion compromise under the extended NPWT assist; this also reduces tissue edema and fluid collection while increasing tissue perfusion.

In our patient series, there was no skin margin necrosis after NPWT-assisted dermatotraction. This method was most effective in cylindrical anatomical area such as trunk and extremities. In these anatomical areas, the fasciotomy wounds were closed directly without tension unless the initial necrotizing fasciitis necrosed the skin flap. Although the skin flap had been involved by the necrotizing fasciitis and partially debrided, NPWT-assisted dermatotraction can decrease the open wound area and minimize donor site morbidity for the secondary operation. Delayed wound dehiscence was observed with Fournier’s gangrene, and the authors thought that inappropriate wound preparation was the primary cause of the failure. However, as Fournier’s gangrene usually occurs at the groin area, its concave contour may lead to inappropriate wound discharge drainage and result in ineffective NPWT-assisted dermatotraction. For the closure of open fasciotomy wounds in necrotizing fasciitis, wound preparation was vital for successful wound closure. We suggest that convex-surfaced cylindrical anatomical areas are more appropriate for NPWT-assisted dermatotraction in the closure of fasciotomy wounds.

Our methods can be applied to fasciotomy wounds after compartment syndrome; however, there are reports of fasciotomy wound closures with dermatotraction alone [9,10]. We think that this type of fasciotomy wound is suppler and less scarred than fasciotomy wounds in necrotizing fasciitis, as it does not require a prolonged period of wound preparation and infection clearance. The authors tried dermatotraction alone for the closure of open fasciotomy wounds in the necrotizing fasciitis, but the scarred, contracted skin flaps were stiff and prone to be macerated or necrosed by the dermatotraction alone. The authors conclude, therefore, that extended NPTW assists mobilization of the scarred open fasciotomy wounds by restoring tissue pressure and eliminating tissue edema.

For better closure of open fasciotomy wounds in necrotizing fasciitis, wound bed preparation and infection clearance are essential. Because increased tissue pressure and wound contraction are affected by extended NPWT decreases over time, timely readjustment and reapplication of extended NPWT-assisted dermatotraction is important in promoting early wound closure.

## Conclusion

Large open wounds after fasciotomies in necrotizing fasciitis patients are difficult to cover. Dermatotraction is an effective treatment option in such patients, but the healing process is extended, and this sometimes results in wound marginal necrosis. The authors applied extended NPWT over dermatotraction simultaneously to facilitate large open fasciotomy wound closure in necrotizing fasciitis. This advances scarred, stiff fasciotomy wound margins synergistically in necrotizing fasciitis, and allows direct closure of the wound without complications. This method can be another good treatment option for the necrotizing fasciitis patient with large open wounds who has poor general condition and is unsuitable for extensive reconstructive surgery.

## Competing interests

The authors declare that they have no competing interests.

## Authors’ contributions

All of the authors were involved in the preparation of this manuscript. JYL participated in the conception, wrote the manuscript and reviewed the literatures. HJ was an assistant surgeon and helped in literature search. HK participated in the clinical and surgical management. SNJ participated in the conception, design of the study, and operated the patient. All authors read and approved the final manuscript.
